# Edema-like symptoms are common in ultra-distance cyclists and driven by overdrinking, use of analgesics and female sex – a study of 919 athletes

**DOI:** 10.1186/s12970-021-00470-0

**Published:** 2021-12-04

**Authors:** Philipp Gauckler, Jana S. Kesenheimer, Andreas Kronbichler, Fiona R. Kolbinger

**Affiliations:** 1 Department of Internal Medicine IV (Nephrology and Hypertension), Medical University Innsbruck, Innsbruck, Austria; 2 Department of Psychology, University of Innsbruck, Innsbruck, Austria; 3 Department of Visceral, Thoracic and Vascular Surgery, University Hospital and Faculty of Medicine Carl Gustav Carus, Technische Universität Dresden, Fetscherstrasse 74, 01307 Dresden, Germany

**Keywords:** Cycling, Ultra-distance cycling, Endurance sports, Exercise-associated hyponatremia, Peripheral edema

## Abstract

**Background:**

Ultra-endurance cyclists regularly report various extents of bodily decline during long-distance bicycle rides, including potential kidney function-related symptoms such as swelling of body parts and urine changes. This study aimed to assess the prevalence of these symptoms in a representative cohort of ultra-endurance cyclists and shed light on potential predictors related to the ride, the rider and the rider’s behavior.

**Methods:**

Between November 26 and December 14, 2020, 1350 people participated in an online survey investigating potential kidney-related symptoms of ultra-distance cycling. Frequency and severity of edema-like (“swelling”) symptoms and perceived changes in urine output, concentration and quality were associated with ride-related factors, demographic parameters and rider behavior-related variables.

**Results:**

A total of 919 participants met the predefined inclusion criteria. The majority (*N* = 603, 65.6%) stated that they suffered from at least one potential kidney function-related symptom, out of which 498 (54.2%) stated one or more edema-like (“swelling”) symptoms. In correlational and multiple regression analyses, female sex, intake of analgesics and drinking strategies correlated with swelling symptoms. Further analyses indicated that drinking due to thirst and/or drinking adapted to ambient sweating and temperature negatively correlated with swelling symptoms, whereas “drinking as much as possible” enhanced these. Intake of analgesics was moderately positively correlated with swelling symptoms.

**Conclusions:**

According to our survey, edema-like symptoms occur in the majority of ultra-distance cyclists and female sex, drinking strategy and intake of analgesic drugs are major predictors thereof. Studies are needed to investigate the underlying pathophysiological processes of such symptoms.

**Supplementary Information:**

The online version contains supplementary material available at 10.1186/s12970-021-00470-0.

## Background

Popularity of ultra-endurance events (e.g. running, swimming and cycling) is rising [1]. Consequently, a growing body of research focuses on health-related issues, ranging from rather inconvenient to potentially life-threatening disturbances that may arise during such extreme activity in otherwise healthy individuals. Additionally, an increasing interest concerning health-related issues in participating endurance athletes yields in a variety of information supply, mostly by non-scientific sources [2]. The vaguely-defined term “ultra-endurance cycling” typically describes bicycle races covering a distance of several hundreds of kilometers, either sub-divided into predefined stages or as single-stage races. While some of these single-stage races allow outside support (e.g. the Race Across America), other races prohibit any forms of outside support that are not equally available to each participant (e.g. the Transcontinental Race/TCR).

Peripheral edema, manifesting as swelling of different body parts including lower (ankles, feet) and upper (hands, fingers) extremities, eyelids, or the entire face, is a symptom that may occur during strenuous exercise. Although early reports of exercise-associated peripheral edema go back to the 1970s [3], respective evidence in the following years is scarce and, in the context of cycling, restricted to very few case reports [4]. Anecdotal reports of different types of swelling symptoms among participants of the TCR and similar ultra-endurance bicycle races have lately been registered in the community, mainly via social media platforms and personal interaction with race participants.

While the term peripheral edema generally describes a condition of excessive fluid accumulation in extracellular spaces of soft tissues, the underlying pathophysiological processes are complex and diverse. The balance of water between the extra- and the intracellular compartments is tightly regulated in the human body. This delicate homeostasis involves the gradient between intra- and extravascular hydrostatic pressures, oncotic pressure differences in plasma and interstitium, the hydraulic permeability of vessel walls, and the lymphatic system, which collects filtered proteins from the interstitium and returns them to the vasculature. Perturbations likely lead to net filtration, resulting in peripheral edema.

The kidney is the central organ to regulate body fluid volume, and peripheral edema can result from reduced plasma oncotic pressure (e.g. urinary loss of proteins) or an increase in capillary hydrostatic pressure (e.g. observed in kidney failure episodes). Continuous sodium and water retention of the kidneys despite expansion of plasma, blood and extracellular fluid volumes is a hallmark of extra-renal causes of peripheral edema [5]. Kidney-mediated fluid homeostasis is tightly regulated by hormones such as antidiuretic hormone (ADH, arginine vasopressin), which is released in response to hyperosmolar conditions to increase water retention. Among ultra-endurance athletes participating in the Southern Traverse adventure race, the plasma volume increased by 25%, and this hypervolemic response strongly correlated with ADH concentrations, while serum sodium levels remained stable [6]. Similar changes in the body composition were found among participants in a 100 km ultra-marathon. An excess of urinary potassium to urinary sodium excretion was observed, and these changes were attributed to an increase in aldosterone levels, a hormone that stimulates water and sodium retention and potassium excretion. Hemodilution was also reported after the Race Across the Alps, covering a total distance of 525 km [7].

The pathogenesis of peripheral edema in otherwise healthy individuals during ultra-endurance exercise is poorly studied. Several hypotheses could potentially explain the development of peripheral edema, including relatively benign alterations such as prolonged elevated capillary hydraulic pressure in a seated position [8], but peripheral edema may also indicate a severe disorder in the otherwise tightly regulated homeostasis of volume and osmosis [9]. To date, peripheral edema has scarcely been reported in endurance sports, mainly in the context of overdrinking (over thirst) and exercise-associated hyponatremia (EAH) [10]. Furthermore, a combination of acute kidney injury (AKI) and exertional rhabdomyolysis due to strenuous exercise may cause peripheral edema [11]. Under these circumstances, the absence of other investigations (e.g. serum creatinine measurements) might mask a potential AKI to chronic kidney disease (CKD) transition, which might be a late sequel of these alterations. However, the discussed hypotheses such as EAH and exertional rhabdomyolysis have mainly been reported in runners and the prevalence in cyclists appears comparably low [7, 11–13].

While a considerable quantity of research focusing on the role of endurance sports on fluid homeostasis is available, little evidence exists on prevalence, pathogenesis, clinical relevance and therapeutic or prophylactic options of peripheral edema in endurance sports in general. Almost no evidence exists on this topic in the context of ultracycling. There is a need to understand the pathophysiology of edema development, the frequency of its occurrence, and potential factors influencing edema onset and severity. Therefore, we conducted a systematic survey assessing both occurrence of peripheral edema and individual practices regarding fluid intake and electrolyte supplementation in ultracyclists.

## Methods

Participants were recruited online through advertisements on social media platforms (Instagram, Facebook, Strava) and a podcast about ultracycling (“*Die wundersame Fahrradwelt”* [the wondrous bicycle world]). The study was introduced as an investigation of “*physical effects of ultracycling [...] In particular, [...] kidney-related symptoms of ultra-endurance bicycle racing*” [14]. The survey was generated using the software “soscisurvey” [15], and was presented to participants online. In addition to demographic parameters including age, gender, education and country of residence, the survey asked participants about manifestation of symptoms such as swelling of body parts or urine changes during one specific “long” bicycle ride, with the definition of “long” remaining up to each participant. Thus, we asked for total kilometers (km) and days covered by this specific bike ride. Answers were coded (Supplementary Information), i.e. “Did you do anything to balance your electrolytes during the race/bike ride?” which was dummy-coded as “1” (if yes) and “0” (if no). The survey asked for “gender” (male, female, diverse), and three participants stated a diverse gender. The question *“What is your gender?”* was selected based on that “proposed online guidance to accompany the sex question in the 2021 [UK] census advises respondents that they may answer in terms of their subjective gender identity, rather than their biological or legal sex” [16]. The cited guidance “assumes that the number of respondents who self-identify as members of the opposite sex will be small, and that the resulting measurement error will therefore be small compared to other sources of misclassification”. To do justice to the fact that biological sex rather than gender identity affects pathophysiological processes, the term “sex” is used throughout this work.

### Participants

One-thousand-three-hundred-and-fifty (*N* = 1350) participants took part in an online survey between November 26 and December 14, 2020. The full survey can be accessed as an OSF (Center For Open Science) online file (DOI 10.17605/OSF.IO/6UEJB). To achieve a highly specific sample, we applied the following inclusion criteria before conducting any analyses: minimum age of 18 years, minimum total distance of the specific ride of 500 km, daily distance between 150 and 1000 km, minimum daily liquid consumption of 1 l, body mass index (BMI) between 15 and 50 kg/m^2^, and maximum total duration of the ride of 30 days.

**Fig. 1:**
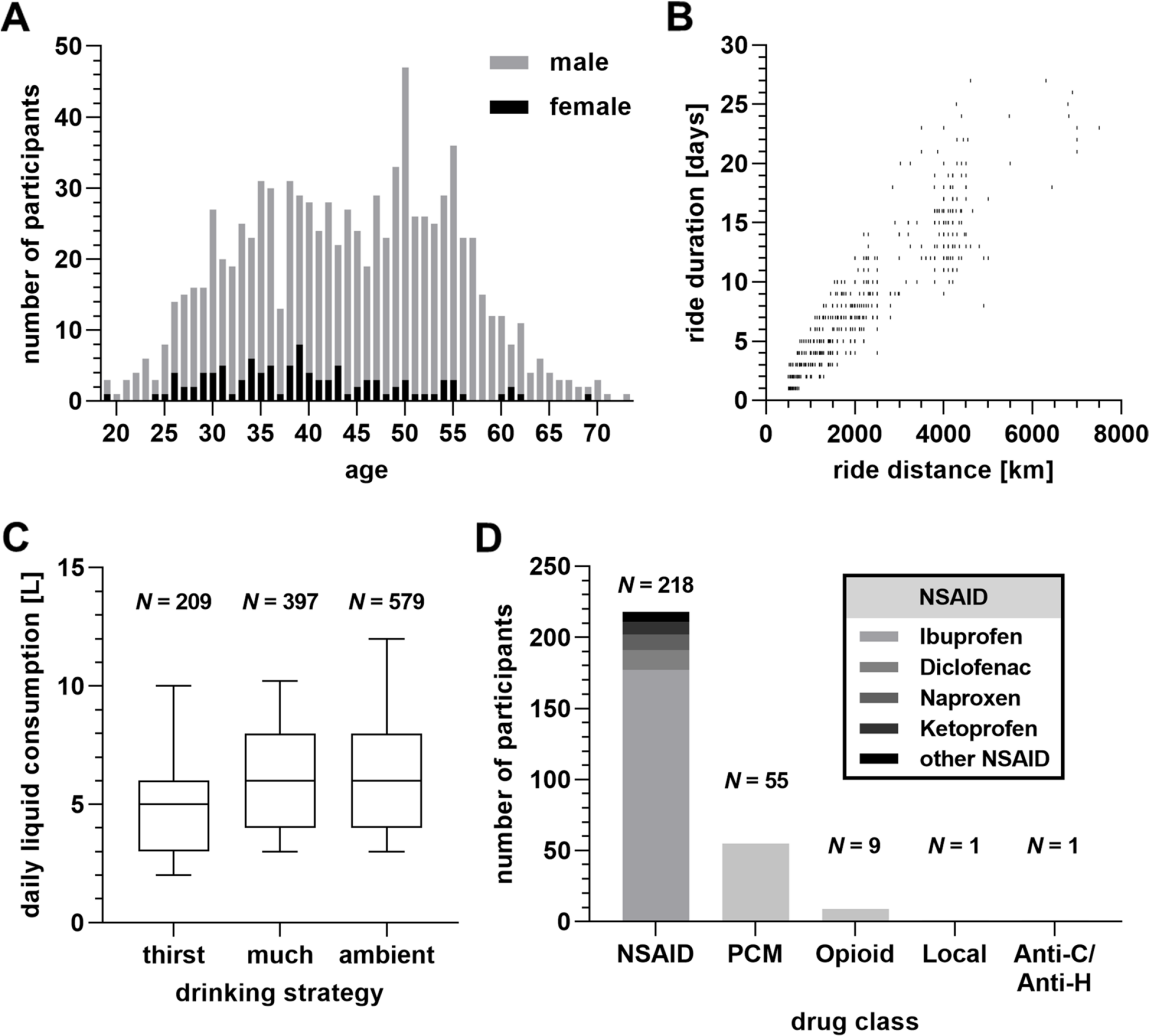
Selected data on the cohort of 919 ultra-endurance cyclists and their reference bicycle rides. **A** Age and sex distribution. Bars indicate total numbers of study participants. **B** Ride distance and duration. Each symbol corresponds to one participant’s reference long-distance bicycle ride. **C** Drinking strategies (thirst: adapted to thirst, much: as much as possible, ambient: adapted to ambient temperature and sweating) and daily liquid consumption during the reference bicycle ride. Boxplots indicate median, interquartile range and 5–95 percentile range. **D** Analgesic intake during the reference bicycle ride. Bars indicate total numbers of study participants. Abbreviations: non-steroidal anti-inflammatory drugs (NSAID), paracetamol (PCM), anticholinergic/antihistamine drugs (Anti-C/Anti-H)

The final sample (*N* = 919, Fig. 1, Tables 1, 2, 3 and 4) consisted of 102 women (11.1%), 814 men (88.6%) and three people stating to be neither male nor female (0.3%). As 695 participants (75.6%) obtained a university degree, the cohort represents an academic sample. Participants were from all over the world (55 countries). Most of them (*N* = 248, 27%) lived in the United Kingdom, Germany (*N* = 178, 19.4%), France (*N* = 68, 7.4%), the United States (*N* = 40, 4.4%), and Belgium (*N* = 39, 4.2%). Demographics of the resulting sample are illustrated in Table 1. Overall, 55.6% (*N* = 511) of participants named an ultracycling race, in which they took part (*N* = 97 Transcontinental Race, *N* = 32 Paris-Brest-Paris, *N* = 16 London-Edinburgh-London, *N* = 14 Trans Pyrenees, *N* = 14 Three Peaks Bike Race).

**Table 1:** The sample’s demographic data

	Overall (*N* = 919)	Female (*N* = 102)	Male (*N* = 814)	Diverse (*N* = 3)
Age	43.95 (10.75; 19–73)	39.73 (9.81; 19–69)	44.48 (10.74; 19–73)	42.00 (14.00; 32–58)
BMI	23.61 (2.51; 17.36–35.51)	22.39 (2.59; 18.65–34.69)	23.78 (2.46; 17.36–35.51)	21.97 (2.22; 20.31–24.97)
Daily distance [km/d] of specific bike ride	306.15 (98.56; 151.25–732.00)	290.90 (97.99; 157.14–650.00)	310.59 (98.52; 151.25–732)	231.11 (42.86; 200.00–280.00)
Duration [d] of specific bike ride	7.10 (5.27; 1–27)	7.58 (5.59; 1–21)	7.04 (5.23; 1–27)	8.33 (5.77; 5–15)
Ultracycling years of experience	5.41 (1.97; 1–8)	4.88 (1.91, 1–8)	5.47 (1.96; 1–8)	5.33 (2.52; 3–8)

*Abbreviations*: *BMI* body mass index, *SD* standard deviation, Cells show: *Mean (SD; min-max)*

**Table 2:** Overall sample characteristics

Symptom	*N*	*%* of total sample	*Mean (SD)* day of onset	*Mean (SD)* severity [1–6 point scale]
Swelling symptoms (overall)	498	54.2%	3.14 (1.56)	2.38 (1.16)
Facial swelling	184	20.0%	3.25 (1.61)	2.20 (1.52)
Eyelid swelling	183	19.9%	3.18 (1.67)	2.22 (1.54)
Swelling of toes/feet	365	39.7%	2.97 (1.56)	2.59 (1.41)
Swelling of fingers/hands	281	30.6%	3.15 (1.67)	2.49 (1.47)
Swelling of extremities (arms/legs)	202	22.0%	3.50 (1.81)	2.39 (1.60)
Urine related symptoms (overall)	524	57.0%	2.22 (1.42)	2.10 (1.04)
Reduced urine output	399	43.4%	2.04 (1.38)	2.52 (1.50)
Increased urine output	105	11.4%	2.58 (1.81)	1.76 (1.23)
Concentrated/darker urine	384	41.8%	2.22 (1.42)	2.62 (1.57)
Less concentrated/lighter urine	89	9.7%	2.67 (1.89)	1.47 (0.92)
Bubbly or foamy urine	53	5.8%	2.91 (1.81)	2.13 (1.71)

*Abbreviations*: *SD* standard deviation

**Table 3:** Female sample characteristics (*N* = 102)

Symptom	*N*	*%* of total sample	*Mean (SD)* day of onset	*Mean (SD)* severity [1–6 point scale]
Swelling symptoms (overall)	80	78.4%	2.76 (1.39)	2.67 (1.14)
Facial swelling	47	46.0%	3.11 (1.54)	2.50 (1.47)
Eyelid swelling	43	42.1%	2.88 (1.49)	2.63 (1.66)
Swelling of toes/feet	59	57.8%	2.56 (1.46)	2.66 (1.30)
Swelling of fingers/hands	56	54.9%	2.87 (1.83)	2.73 (1.40)
Swelling of extremities (arms/legs)	41	40.2%	3.12 (1.72)	2.81 (1.66)
Urine related symptoms (overall)	72	70.6%	2.17 (1.10)	2.43 (1.28)
Reduced urine output	62	60.8%	1.96 (1.25)	2.88 (1.66)
Increased urine output	16	15.6%	3.07 (1.77)	2.06 (1.46)
Concentrated/darker urine	50	49.0%	1.98 (1.02)	2.84 (1.67)
Less concentrated/lighter urine	14	13.7%	2.85 (1.82)	1.71 (1.06)
Bubbly or foamy urine	6	5.9%	2.67 (1.51)	2.69 (1.98)

*Abbreviations*: *SD* standard deviation

**Table 4:** Male sample characteristics (*N* = 814)

Symptom	*N*	*%* of total sample	*Mean (SD)* day of onset	*Mean (SD)* severity [1–6 point scale]
Swelling symptoms (overall)	416	51.1%	3.21 (1.57)	2.33 (1.15)
Facial swelling	135	16.6%	3.26 (1.61)	2.15 (1.53)
Eyelid swelling	138	16.9%	3.24 (1.69)	2.15 (1.51)
Swelling of toes/feet	304	37.3%	3.05 (1.57)	2.58 (1.44)
Swelling of fingers/hands	224	27.5%	3.21 (1.62)	2.46 (1.48)
Swelling of extremities (arms/legs)	160	19.7%	3.59 (1.83)	2.32 (1.58)
Urine related symptoms (overall)	451	55.4%	2.23 (1.47)	1.05 (0.99)
Reduced urine output	336	41.2%	2.01 (1.40)	2.47 (1.47)
Increased urine output	89	10.9%	2.50 (1.81)	1.71 (1.18)
Concentrated/darker urine	333	40.9%	2.19 (1.47)	2.59 (1.55)
Less concentrated/lighter urine	74	9.1%	2.64 (1.94)	1.43 (0.89)
Bubbly or foamy urine	47	5.8%	2.95 (1.87)	2.05 (1.65)

*Abbreviations*: *SD* standard deviation

### Statistical analyses

#### Correlations and differences between groups

Normal distribution was denied for all symptoms according to Shapiro-Wilk tests (*p* < .05). Therefore, non-parametric raw correlations were used, which are reported in Tables 5 and 6.

**Table 5:** Spearman rho correlations

	Ride distance [km]	Ride duration [d]	Daily distance [km/d]	Sex (f = 1, m = 2)	BMI [kg/m^2^]	Electrolyte intake (no = 0, yes = 1)	Fluid intake [L/d]	Drinking strategy			Analgesic intake (no = 0, yes = 1)
								ambient	much	thirst	
Swelling Symptoms (total)	.06 [−.02, .14]	.06 [−.02, .14]	−.03 [−.11, .06]	−.22*** [−.29, .13]	.06 [−.02, .14]	−.02 [−.11, .08]	−.02 [−.10, .06]	−.07 [−.15, .92]	.11** [.03, .19]	−.05 [−.14, .04]	.20*** [.12, .28]
Facial Swelling	.09* [.01, .17]	.09* [−.00, .17]	−.02 [−.10, .07]	−.21*** [−.30, −.12]	−.11** [−.19, −.03]	−0.03 [−.12, .05]	.01 [−.08, .10]	−.01 [−.08, .07]	.04 [−.04, .11]	−.02 [−.09, .07]	.11** [.02, .18]
Eyelid Swelling	.08* [.01, .16]	.07(*) [−.01, .16]	.01 [−.07, .08]	−.18*** [−.27, .08]	−.11* [−.19, −.04]	.02 [−.06, .10]	−.02 [−.10, .05]	.02 [−.07, .10]	.07(*) [−.00, .15]	−.05 [−.13, .03]	.10* [.02, .18]
Swelling of toes/feet	.04 [−.04, .11]	.02 [−.06, .10]	.01 [−.07, .08]	−.06 [−.14, .03]	.05 [−.03, .12]	.01 [−.08, .09]	−.00 [−.07, .08]	−.05 [−.13, .03]	.08* [.01, .17]	−.05 [−.14, .03]	.18*** [.10, .25]
Swelling of fingers/hands	−.03 [−.11, .05]	−.02 [−.10, .06]	−.02 [−.11, .06]	−.14** [−.22, .06]	.08* [.00, .15]	−.06 [−.14, .03]	−.00 [−.09, .07]	−.09* [−.17, −.01]	.12* [.04, .20]	−.03 [−.11, .05]	.12* [.04, .20]
Swelling of extremities	.05 [−.04, .13]	.05 [−.04, .12]	−.03 [−.11, .05]	−.13* [−.22, .04]	−.03 [−.10, .05]	−.02 [−.10, .06]	−.01 [−.08, .07]	−.05 [−.14, .02]	.07(*) [−.01, .16]	−.04 [−.12, .04]	.12* [.04, .20]
Reduced urine output	−.16*** [−.23, −.07]	−.12** [−.19, −.04]	−.02 [−.09, .07]	−.06 [−.14, .02]	−.00 [−.09, .08]	.04 [−.04, .13]	.01 [−.07, .09]	−.03 [−.11, .05]	.05 [−.03, .13]	.03 [−.04, .12]	.01 [−.07, .09]
Increased urine output	.09* [.01, .18]	.08* [−.00, .16]	−.00 [−.08, .08]	−.03 [−.11, .06]	−.06 [−.14, .01]	.05 [−.03, .11]	.05 [−.03, .13]	−.00 [−.08, .08]	−.01 [−.08, .07]	−.07 [−.15, .01]	.02 [−.05, .10]
Concentrated/darker urine	−.12** [−.21, −.04]	−.10* [−.17, −.02]	−.01 [−.09, .06]	.03 [−.10, .12]	.08* [−.00, .16]	.07 [−.03, .16]	−.05 [−.13, .03]	−.13** [−.21, −.05]	.04 [−.04, .12]	.08(*) [−.01, .16]	−.01 [− 10, .06]
Less concentrated/lighter urine	.02 [−.06, .09]	.01 [−.07, .09]	.01 [−.08, .10]	−.02 [−.10, .06]	−.04 [−.12, .05]	−.03 [−.11, .05]	.09* [.02, .17]	.01 [−.07, .09]	.03 [−.05, .11]	−.07(*) [−.14, .01]	.02 [−.06, .10]
Bubbly/Foamy urine	−.01 [−.09, .08]	−.02 [−.10, .06]	.01 [−.07, .09]	.02 [−.06, .08]	−.03 [−.11, .06]	.05 [−.02, .09]	−.03 [−.12, .04]	−.05 [−.14, .03]	−.03 [−.11, .05]	.06 [−.03, .15]	.04 [−.04, .13]

Parentheses indicate 95% confidence intervals. Levels of significance: (*): marginal significance *p* < .10, *: *p* < 0.05, **: *p* < 0.01, ***: *p* < 0.001. Abbreviations: body mass index (BMI)

**Table 6:** T values of linear multiple regression models

	*df*	Ride distance [km]	Ride duration [d]	Daily distance [km/d]	Sex (f = 1, m = 2)	BMI [kg/m^2^]	Electrolyte intake (no = 0, yes = 1)	Fluid intake [L/d]	Drinking strategy			Analgesic intake (no = 0, yes = 1)
									ambient	much	thirst	
Swelling symptoms (overall)	591	1.24	−.96	−.40	−5.26***	−.20	−.43	.49	−.55	2.11*	−.31	4.28***
Women	75	.72	−.20	.43		.93	1.12	−.53	−.85	−.77	−.05	1.20
Men	504	1.11	−.99	−.51		−.50	−.76	.64	.01	2.61**	−.32	3.72***
Facial swelling	593	.22	.44	.82	−3.91***	−.91	.37	.51	−.08	.73	−1.21	2.06***
Women	75	.94	−.43	−.00		.62	1.87(*)	.36	−.71	−.53	.11	.61
Men	506	−.14	.67	.93		−.88	−.29	.20	.29	.83	−1.45	1.60
Eyelid swelling	593	1.12	−.59	.14	−3.67***	−.61	.39	−.41	.15	1.17	−1.69(*)	2.08*
Women	75	1.06	−.56	−.12		−.44	.99	−.36	−.21	−.83	−.14	1.09
Men	506	.80	−.41	.23		−.33	−.04	−.44	.34	1.46	−1.80(*)	1.50
Swelling of fingers/hands	593	−.14	.06	.01	−2.86**	2.04*	−.44	.52	−.63	1.79(*)	−1.37	2.31*
Drinking strategy*BMI	590	−.04	.00	−.01	−2.90**	.26	−.45	.63	1.35	−.05	−1.36	2.25*
Women	72	.28	.26	−.32		.00	.52	.05	.24	−.44	−.18	.01
Men	503	.04	−.20	.04		−.08	−.45	.63	1.33	−.05	−2.03*	2.07*
Swelling of toes/feet	593	.54	−.32	−.02	−1.23	1.10	.72	−.09	−.57	1.02	−1.74	3.72***
Swelling of extremities (arms/legs)	593	.72	−.56	−.63	−2.55*	−.33	.12	.85	−.92	.38	−2.03*	2.26*
Women	75	.90	−.78	.76		1.54	−.03	−1.03	.41	−.43	−.80	.65
Men	506	.06	.07	−.52		−1.04	.53	1.23	−1.02	.65	−1.79(*)	2.14*
Reduced urine output	593	−.64	−.04	.13	−1.32	.03	1.37	.63	.31	1.63	−.08	.99
Increased urine output	593	.60	−.01	.09	−.79	−.48	1.98*	.31	−1.3	−1.23	−2.85**	1.02
Concentrated/darker urine	593	.33	−.71	−.42	.32	1.08	2.41*	−.58	−2.00*	.71	.59	.70
Less concentrated/lighter urine	593	−.23	.44	.63	−.76	.44	2.25*	1.04	−.54	.02	−2.43*	.91
Bubbly/foamy urine	593	.86	−.64	−.39		.40	.88	.06	−.76	−.14	−1.39	.94

Indented: specification of model, first column: dependent variable, second column: degrees of freedom, third column: predictors. Levels of significance: (*): marginal significance p < .10, *: *p* < 0.05, **: *p* < 0.01, ***: *p* < 0.001. Abbreviations: body mass index (BMI)

#### Regression analyses

To analyze dependencies between variables, multiple linear regression models were conducted. Here, occurrence of symptoms served as a dependent variable. The following variables were taken into account as predictors: total distance of the ride [km], total duration of the ride [days], daily distance [km/day], BMI, electrolyte intake, sex, daily liquid consumption (liters), liquid intake habits (ambient, thirst, as much as possible) and intake of analgesics. In a first step, regression models with only main effects of predictors were investigated. In a second step, if BMI was a significant predictor, an interaction effect of BMI and drinking behavior (ambient*BMI, thirst*BMI and much*BMI) was added. In a third step, regressions were modeled separately for women and men in order to investigate differences between sexes. Due to the low sample size of three people stating diverse sex, these participants were excluded from sex-specific analyses.

## Results

### Prevalence of symptoms

About two thirds of participants (*N* = 603, 65.6%) stated that they suffered from at least one of the following symptoms: facial or eyelid swelling, swelling of toes/feet or fingers/hands, swelling of extremities (arms or legs), reduced or increased urine output, concentrated or less concentrated urine, bubbly or foamy urine. Four-hundred-and-ninety-eight (*N* = 498, 54.2%) participants stated at least one swelling symptom, and 524 (57.0%) stated at least one urine-related symptom. On average, swelling symptoms and urine-related symptoms onset after 3.14 (± 1.56) days and 2.22 (± 1.42) days of the bicycle ride, respectively. Details on the prevalence of specific symptoms are outlined in Tables 2, 3, and 4.

The question about weight changes pre to post bike ride was answered by 644 participants (70.1%). Out of these, 37 participants who weighed themselves had gained weight and another 25 participants who did not weigh themselves felt they gained weight (total *N* = 62, 6.8%). Most participants lost weight or felt they lost weight (*N* = 582, 63.3%).

### Predictors of edema (1): female sex correlates with edema-like symptoms

**Fig. 2:**
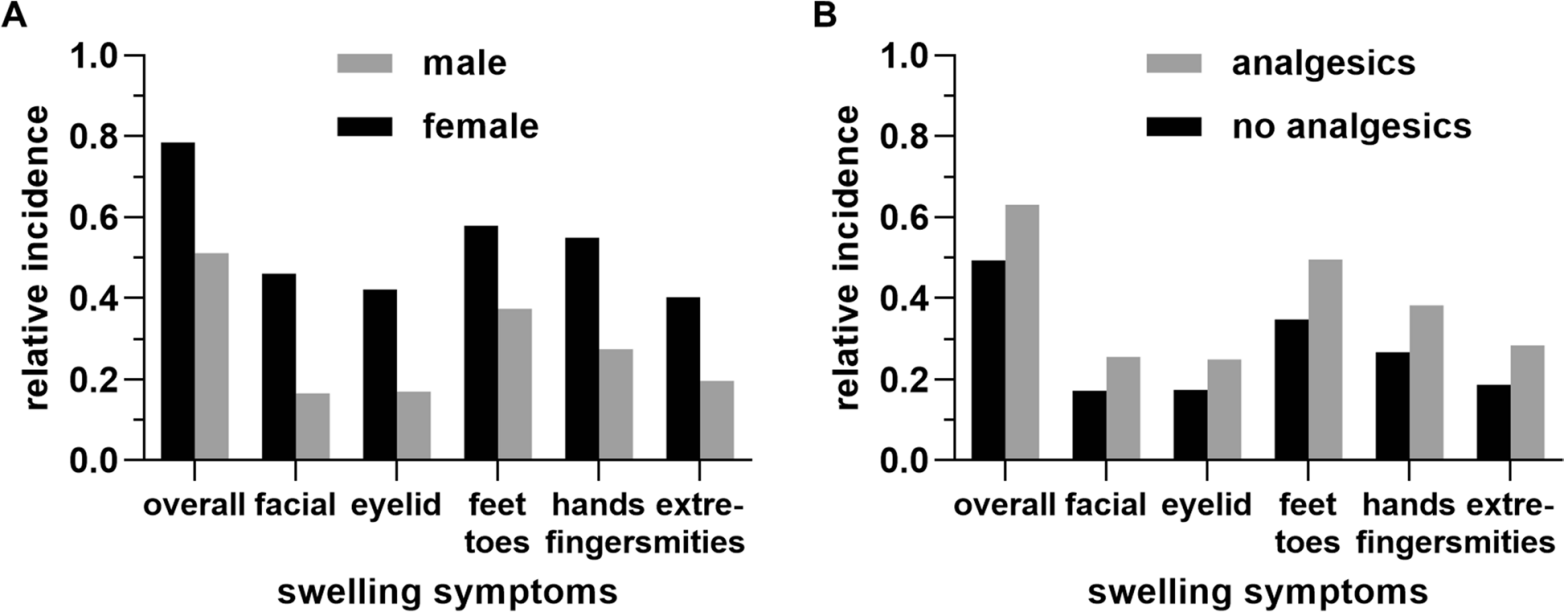
Incidence of swelling symptoms during the participants’ reference bicycle rides depending on sex (**A**) and analgesic intake (**B**). Bars indicate relative incidences

Overall, women suffered from swelling symptoms more frequently than men (Fig. 2 A). These differences between men and women were significant in terms of the following symptoms: facial swelling (*T*(1, 599) = 6.05, *p* < .001), eyelid swelling (*T*(1, 599) = 5.24, *p* < .001), swelling of fingers/hands (*T*(1, 599) = 3.15, *p* = .002) and swelling of extremities (*T*(1, 599) = 3.54, *p* < .001; Fig. 2 A). Other symptoms did not show significant differences between female and male participants (Tables 2, 3 and 4).

Linear regression models (Table 6) confirmed the results of the raw correlations (Table 5). Importantly, there were no sex differences in drinking strategies (ambient: *T*(914) = − 1.19, *p* = .234; thirst: *T*(914) = .962, *p* = .336; as much as possible: *T*(914) = −.632, *p* = .527). Men and women did not differ in estimated daily fluid intake (*Mwomen* = 5.67, *Mmen* = 6.02, *t*(121) = − 1.04, *p* = .296), but differed in terms of BMI *(t*(125) = − 5.12, *p* < .001). Women had a slightly lower BMI (*Mwomen* = 22.39) than men (*Mmen* = 23.78). However, as BMI was no significant predictor for any symptom (Tables 5 and 6), these differences were not considered further.

With regard to body mass-normalized daily fluid intake, men and women differed significantly. Women drank an estimated amount of 0.092 (± 0.057) liters per kg body weight per day, while men drank 0.078 (± 0.036) liters per kg (*t*(111) = 2.41, *p* = .018). However, ingested fluid amount per kg body weight neither predicted swelling symptoms in the linear regression model (*t*(590) = −.34, *p* = .734), nor urine-related symptoms (*t*(590) = 1.15, *p* = .253). Meanwhile, other relations, especially the influence of sex, remained stable in these analyses. Therefore, we did not further investigate fluid intake per body weight as a relevant covariate.

### Predictors of edema (2): drinking strategies are related to edema-like symptoms

In total, 63% (*N* = 579) of participants affirmed that they adapted their liquid intake to ambient temperature and intensity of sweating. However, 43.2% (*N* = 397) stated that they drank as much as possible. Another 22.7% (*N* = 209) affirmed they only drank when they were thirsty. Only 0.3% (*N* = 3) of participants affirmed that they drank “*as little as possible to reduce weight*” (Fig. 1 C).

Drinking adapted to ambient temperature and sweating negatively correlated with swelling of fingers and hands and concentrated/darker urine. Further, drinking as much as possible positively correlated with overall swelling symptoms, the swelling of fingers and hands as well as toes and feet. However, drinking behavior correlated with BMI. The correlation of BMI and “*drinking as much as possible*” was *r* = .09 ([.03, .15], *p* = .009), while the correlation of BMI and drink “*adapted to ambient*” was *r* = −.09 ([−.16, −.03], *p* = .005). Additionally, there was a marginal significant correlation of BMI and “*drink only when thirsty*”, *r* = .06 ([−.01, .12], *p* = .089). This is why we included an interaction effect of BMI and drinking strategies into linear regression models, whenever BMI was a significant predictor.

Drinking as much as possible positively predicted overall swelling symptoms for men, and marginally, the swelling of fingers and hands. Only drinking when thirsty negatively predicted the following symptoms: eyelid swelling (marginally), swelling of fingers and hands in men, and swelling of extremities (arms/legs). Additionally, only drinking due to thirst was negatively related to increased urine output and less concentrated/lighter urine. Drinking adapted to ambient temperature negatively predicted concentrated/darker urine. The estimated liquid intake per day did not have any effects on dependent variables (Tables 5 and 6).

### Predictors of edema (3): intake of analgesics correlates with edema-like symptoms

Two-hundred-and-sixty (*N* = 260; 28.3%) participants took analgesics due to pain during the specific long distance bike ride. In addition, sixty-nine (*N* = 69, 7.5%) participants stated that they took analgesics preventively (before pain occurred). The remaining participants (*N* = 610, 66.4%) stated that they took no analgesics at all. The most frequently used analgesics were non-steroidal anti-inflammatory drugs (NSAIDs, *N* = 218), paracetamol (*N* = 55), and opioids (*N* = 9) (Fig. 1 D). While the use of analgesics was unrelated to urine-related symptoms, it moderately correlated with swelling symptoms. In particular, use of analgesics correlated with facial swelling, eyelid swelling, swelling of toes and feet, fingers and hands, and of extremities (Table 5, Fig. 2 B). Linear multiple regression analyses showed that use of analgesics was positively related to all swelling symptoms, but not to urine-related symptoms (Table 6).

### Predictors of edema (4): electrolyte intake does not correlate with edema-like symptoms

In regression models, electrolyte intake was positively related to increased urine output, darker *and* lighter urine, but unrelated to swelling symptoms (Table 6). Comparison of participants who took electrolytes and participants who did not, as well as correlational analyses (Tables 5 and 6), did not identify any relations (swelling symptoms, overall: *T*(1, 601) = .631, *p* = .528; facial swelling: *T*(1, 601) = .603, *p* = .547; eyelid swelling: *T*(1, 601) = −.539, *p* = .590; swelling of toes/feet: *T*(1, 601) = −.061, *p* = .951; swelling of fingers/hands: *T*(1, 601) = 1.41, *p* = .159; swelling of extremities: *T*(1, 601) = .524, *p* = .601; reduced urine output: *T*(1, 601) = − 1.06, *p* = .289; increased urine output: *T*(1, 601) = − 1.48, *p* = .140; concentrated/darker urine: *T*(1, 601) = − 1.43, *p* = .152; less concentrated/lighter urine: *T*(1, 601) = .496, *p* = .620 and bubbly/foamy urine: *T*(1, 601) = − 1.28, *p* = .201).

### Predictors of edema (5): intake of contraceptives does not correlate with edema-like symptoms

Twenty-six (2.8% of the total sample; twenty-five female, one diverse) took hormonal contraceptives at the time of the specific long bicycle ride. Twenty-four of those participants using hormonal contraceptives claimed any kind of symptom. Although groups of participants taking hormonal contraceptives and participants not doing so were naturally unequally sized, additional analyses were performed to gain further insights in the role of hormonal contraceptives for developing swelling symptoms. First, the group taking contraceptives and the group not taking contraceptives were compared using a t-test for independent samples. Results showed a significant difference for facial swelling (*t(*596) = − 2.05, *p* = .041) and eyelid swelling (*t*(596) = − 2.27, *p* = .024) only, with participants taking contraceptives claiming higher prevalence of swelling symptoms than the others (facial: *M* = 1.71 (*SD* = .15) vs. *M* = 1.34 (*SD* = .04); eyelid: *M* = 1.75, (*SD* = .17) vs. *M* = 1.34 (*SD* = .04). For all other symptoms, there were no significant differences.

However, when further investigating the role of contraceptives in the interplay with other variables, these differences were not maintained in multiple linear regression analyses. Predicting facial swelling, the intake of contraceptives was no longer influential (*β* = .00, *t*(585) = .03, *p* = .973), when simultaneously taking daily distance, duration of the bike ride, BMI, electrolyte intake, sex, drinking strategies and analgesic intake into account (model statistics: *F*(585) = 3.01, *p* < .001). Similarly, use of hormonal contraceptives was not influential for predicting eyelid swelling in linear multiple regression analysis (*β* = .00, *t*(585) = .50, *p* = .618; model statistics: *F*(585) = 2.88, *p* < .001). All results of these multiple regression models are summarized in Table 7.

**Table 7:** T values of the linear multiple regression model including contraceptive intake

	*df*	Ride distance [km]	Ride duration [d]	Daily distance [km/d]	Sex (f = 1, m = 2)	BMI [kg/m^2^]	Electrolyte intake	Fluid intake [L/d]	Drinking strategy			Analgesic intake (no = 0, yes = 1)	Contraceptives intake (no = 0, yes = 1)
									ambient	much	thirst		
Facial Swelling	585	.19	.48	.78	−3.54**	−.87	.26	.67	−.17	.80	−1.19	1.82	.03
Eyelid Swelling	585	1.02	−.51	.15	−3.04**	−.60	.42	−.34	.13	1.18	−1.64	2.04*	.50

Indented: specification of model, first column: dependent variable, second column: degrees of freedom, third column: predictors. Levels of significance: (*): marginal significance p < .10, *: p < 0.05, **: p < 0.01. Abbreviations: body mass index (BMI)

### Predictors of edema (6): menopause does not correlate with edema-like symptoms

Menopause usually starts around the age of 50 [17]. To investigate the effects of menopause, we compared women younger (*N* = 86) and older (*N* = 16) than the age of 50. The following results need to be considered in awareness of the rather small sample size of menopaused women (Fig. 1). In a t-test for independent samples, no significant differences were found; women over the age of 50 did not differ from other female participants in terms of any potential kidney-related symptom.

## Discussion

To our knowledge, no study has systematically investigated the symptom of peripheral edema in ultra-distance cyclists to date. In this survey study of 919 ultra-distance cyclists, over half of the participants (54.2%) reported experience of edema-like (“swelling”) symptoms during or after a long-distance bike ride. While cardiac symptoms such as heart rate alterations [18] or the presence of cardiac damage biomarkers in the bloodstream [19] have been reported previously, peripheral edema has not been described in ultra-endurance cyclists previously. Given that the mean onset of edema-like symptoms was after 3.14 days in our cohort, respective symptoms may not have occurred in studies on shorter races [7]. Swelling symptoms in all individual body parts (face, eyelid, fingers/hands, toes/feet, extremities) were more prevalent in female participants. After regression analyses, drinking behavior (“drinking as much as possible”) and intake of analgesics independently predicted occurrence of edema in men.

### Potential pathophysiology (1): EAH

Considering drinking behavior as an independent risk factor of edema-like symptoms in our cohort, EAH, defined as a plasma sodium concentration < 135 mmol/L during or after exercise, could contribute to this phenomenon. Although EAH is mostly diagnosed in oligo- or asymptomatic patients, severe hyponatremia (< 120 mmol/L) can cause cerebral edema, making this electrolyte disorder a potentially life-threatening condition. Current evidence suggests that EAH is caused by a combination of excessive fluid intake (drinking over thirst) and inadequate suppression of antidiuretic hormone (ADH) secretion [20]. Female sex is a recognized risk factor for EAH but the connection is not clearly explained [20]. A higher fluid intake in women was shown under laboratory conditions [21] but could not be verified in respective field studies [22–24]. Other authors assumed a lower body weight rather than female sex itself as the major explanation for higher EAH rates in women [25]. Given that in our cohort, only female sex but not BMI or fluid intake correlated with the development of swelling symptoms, we assume a sex-specific effect predisposing women for edema-like symptoms, independent of drinking behavior and BMI. Susceptibility of women for EAH may be explained by elevated levels of estradiol and progesterone which have been shown to be associated with fluid retention and sodium loss in premenopausal women [24]. As hyponatremia results from plasma dilution due to fluid overload in EAH, current recommendations suggest to avoid overdrinking during endurance activities [20, 26]. In this context, reported drinking strategies in our cohort are alarming, as 43.2% (*N* = 397) stated to drink as much as possible. Additional risk factors for the manifestation of EAH such as the use of NSAIDs are discussed [20].

In our cohort, about one third of all participants stated to take analgesics during the race either to treat pain (28.3%) and/or preventively (7.5%), and intake correlated significantly with the occurrence of edema-like symptoms. Considering the observational study design, no conclusion on causality of this association can be made.

### Potential pathophysiology (2): exertional rhabdomyolysis

In ultra-distance cyclists, the cause of kidney injury remains elusive. Exertional rhabdomyolysis, a condition associated with both heavy exercise and kidney injury as a result of excessive myoglobin release into the bloodstream, has been described primarily in ultra-endurance runners [12]. One explanation for the low rate of rhabdomyolysis seen in cyclists is the mainly concentric muscular exercise with little eccentric muscular exercise, which is mainly responsible for skeletal muscle damage [27]. Two studies investigating kidney function during single-day marathon cycling races over 230 km and 525 km identified a transiently mildly decreased creatinine clearance. Neither of these studies reported any case of exertional rhabdomyolysis. Of note, no female athlete was included [7, 28].

### Potential pathophysiology (3): Hypoproteinemic edema

A third potential cause of peripheral edema in ultra-distance cyclists is hypoproteinemic edema due to a severely catabolic metabolism. Bircher et al. reported the case of a 34-year-old male ultra-endurance cyclist who lost 2.0 kg of body mass during a 2272 km cycling race [4]. Another study examined 36 participants of the Swiss cycling marathon (600 km distance) and found a mean loss of 1.7 kg body mass and 1.4 kg fat mass without significant loss of skeletal muscle mass [29]. In these athletes, circumferences of the lower extremities decreased, skinfold thickness of the lower limbs increased, which might explain “swelling” symptoms as reported in our cohort. It remains to be elucidated if degradation of muscle mass and hypoproteinemia only set in after several days of cycling and could thus be a potential cause of edema. In ultratriathlon, e.g. triple triathlon or ten-time triathlon, or in ultra-marathon a reduction of skeletal muscle mass has been reported repeatedly [30–32].

### Potential pathophysiology (4): alternative explanations

Fluid dynamics during prolonged exercise differ between women and men. In ninety-eight participants walking between 30 and 50 km, men demonstrated a larger decrease in body mass and a higher incidence of dehydration. This could be explained by a higher fluid loss (5.0 mL/kg/h) and a lower fluid intake (2.9 mL/kg/h) in comparison to women (4.8 and 3.7 mL/kg/h), respectively [33]. This might be explained by differences in osmoregulation between men and women, characterized by greater sensitivity in plasma arginine vasopressin response to changes in plasma osmolality [34]. Fluid intake was not only found to differ during longer periods of exercise [33], but also in elderly people during four bouts of 15 min cycling [21]. On a per kilogram basis, fluid intake was significantly higher among women compared to men. This could, in part, explain the sex differences observed in our survey, but quantitative analyses of fluid intake assessed in a retrospective survey form needs to be interpreted with caution. Drinking strategies did not differ between both sexes in our study. Body composition differs between men and women and a steady change is observed during the span of life. Both sexes have an increase in waist and waist-to-hip ratio with age, and this is partly independent of increases in BMI alone [35]. The age-associated decrease in lean mass and an increase in body fat was higher among women in a population-based study from Korea [36], further highlighting that addition of body composition measures to other parameters seems critical when assessing the risk of “swelling”.

Analgesic intake moderately correlated with the occurrence of “swelling symptoms” in our analysis. The frequency of edema in large trials investigating the use of NSAIDs in general ranged between 1.9 to 6.3%. Evidence suggests that this symptom similarly occurs with all classes used (i.e. selective and non-selective cyclooxygenase inhibitors) and is reversible after discontinuation [37]. Chronic NSAID use impairs hemodynamic capacities of the kidney, and thus might prone athletes to develop kidney-related symptoms. Future research needs to address these considerations and needs to expand research to female ultra-distance cyclists, as there is increasing popularity among women and female participants were underrepresented in past research efforts.

### Limitations and strengths

The main limitation of our study is its strictly descriptive design and presence of biases as typically seen in survey studies (e.g. response bias due to low response rates, recall bias or data inconsistency). Therefore, a particular effort was made to face these biases accordingly during different study phases, including study design, survey development, statistical analysis and interpretation of results [38]. Moreover, the specific wording of the questions and the emphasis of the survey on in-depth analysis of the incidence, onset and risk factors for potential kidney function-related symptoms could have introduced bias. While entirely open questions could possibly have resulted in more general findings on the incidence of any symptoms of ultra-distance cycling, this focus on a subset of symptoms was necessary for generation of hypotheses about the pathogenesis of potential kidney function-related symptoms such as urine changes and edema.

With regard to demographics, the survey asked for “gender” (male, female, diverse), and three participants stated a diverse gender. While the discrepancy between “gender” and “sex” is likely negligible and not statistically relevant in the context of this research [16], the survey did not specifically ask participants for their biological sex, introducing the chance of misinterpretation based on potential discrepancies between biological sex and sociocultural gender identity.

In order to obtain reliable responses, we paid attention to keep the survey as compact as possible, use unambiguous and simple questions and colloquial speech (e.g. “painkiller”) instead of technical terms (“analgesics”) wherever possible. With 919 participants meeting the inclusion criteria and over 10% female athletes we believe this cohort reflects a notable and representative sample and to our knowledge represents the largest cohort of ultra-endurance cyclists in literature to date.

The report of “swelling” was a subjective impression by the athletes. Subjective limb swelling, e.g. due to vasodilatation, cannot be distinguished from true peripheral edema by our survey. Furthermore, study participants reported a wide BMI range, which might also influence the occurrence of “swelling symptoms”. Considering that the reported mean BMI was within an expected range and standard deviations acceptable, this confounder appears minor.

Given the high prevalence of edema-like symptoms in ultra-endurance cyclists identified in this study and the diversity of potential underlying mechanisms, a study objectivizing associated changes in kidney function should be conducted. Such a comprehensive clinical evaluation should aim at considering all mechanisms that may contribute to fluid homeostasis, including the cardiovascular system, fluid and nutrient intake, and fluid homeostasis mediated by the kidneys and hormones. Ultimately, this could result in recommendations for kidney-protective behaviors during ultra-endurance cycling.

## Conclusions

Edema-like symptoms are common in ultra-distance cyclists, and female sex, intake of analgesic drugs and reported maximization of liquid intake positively correlate with the occurrence of edema-like symptoms.

The underlying pathophysiology of peripheral edema remains unresolved and existing potential explanations in current literature do not satisfactorily explain the high rates of edema-like symptoms observed in our cohort. Both EAH and exertional rhabdomyolysis are rarely described in ultra-distance cyclists and not typically associated with peripheral edema. Hypoproteinemic edema may explain the late onset of symptoms after > 3 days but available literature is conflictive and scarce.

Potentially harmful behaviors such as intake of analgesic drugs and maximization of liquid intake are relevant issues among ultra-distance cyclists and respective educational work is required.

## Supplementary Information

**Additional file 1.** Supplementary material.

## Data Availability

The datasets generated and analyzed during the current study are available from the corresponding author on reasonable request.

## Abbreviations

ADH: Antidiuretic hormone
AKI: Acute kidney injury
Anti-C/Anti-H: Anticholinergic/antihistamine drugs
BMI: Body mass index
CKD: Chronic kidney disease
EAH: Exercise-associated hyponatremia
NSAID: Non-steroidal anti-inflammatory drugs
PCM: Paracetamol
SD: Standard deviation
TCR: Transcontinental Race
