# Supplementary material for: Edema-like symptoms are common in ultra-distance cyclists and driven by overdrinking, use of analgesics and female sex – a study of 919 athletes
Source: J Int Soc Sports Nutr. 2021 Dec 4;18:73. doi: 10.1186/s12970-021-00470-0 (PMC8643017; doi:10.1186/s12970-021-00470-0)
Supplement: Supplementary file 1 — Additional file 1. Supplementary material. [file 12970_2021_470_MOESM1_ESM.docx]

**Supplementary material**

**Coding**

Women were coded by “1”, men by “2” and diverse by “3”.  Due to low sample sizes of diverse participants (*N* = 3), these cases were not considered in the investigation of sex differences. Electrolyte intake was assessed by the question “*Did you do anything to balance your electrolytes during the race/bike ride?*”. Answers were dummy-coded as “1”, if yes and “0” if no. Drinking habits were assessed by single items: “*Adapt liquid intake to ambient temperature and intensity of sweating*” (ambient), “*Only drink when I am thirsty*” (thirst), “*I try to drink as much as possible*” (much), and “*As little as possible to reduce weight*” (little). Answers were coded by “1” if chosen and by “0” otherwise. Intake of analgesic drugs (“painkiller intake”) was dummy-coded by “1” if painkillers were taken and “0” if not. In addition, participants named the drugs they took during the bike ride.

In particular, we asked participants if they suffered from the following symptoms: facial swelling, eyelid swelling, swelling of toes/feet or fingers/hands, swelling of extremities (arms or legs), reduced or increased urine output, concentrated or less concentrated urine and bubbly or foamy urine. For each symptom, answers were either “not at all” (coded as “1”), “a bit” (coded as “2”) or “very much” (coded as “3”). Additionally, we asked for the symptoms’ onset (“not at all”, “day 1”, ... to “day 7 or later”, “after the race”). “Overall swelling symptoms" and “overall urine-related symptoms", respectively, were coded by a sum score of all swelling symptoms.
